# Cytokines and their regulators in rat lung following scorpion envenomation

**DOI:** 10.1016/j.toxcx.2024.100198

**Published:** 2024-04-08

**Authors:** Valery Gunas, Oleksandr Maievskyi, Tatyana Synelnyk, Nataliia Raksha, Tetiana Vovk, Tetiana Halenova, Olexiy Savchuk, Igor Gunas

**Affiliations:** a Department of Biochemistry, Educational and Scientific Center Institute of Biology and Medicine, Taras Shevchenko National University of Kyiv, Kyiv, Ukraine; b Department of Clinical Medicine, Educational and Scientific Center Institute of Biology and Medicine, Taras Shevchenko National University of Kyiv, Kyiv, Ukraine; c Department of Human Anatomy, National Pirogov Memorial Medical University, Vinnytsya, Ukraine; d Department of Forensic Medicine and Law, National Pirogov Memorial Medical University, Vinnytsya, Ukraine

## Abstract

Nowadays, more than two billion inhabitants of underdeveloped tropical and subtropical countries are at risk of being stung by scorpions. Scorpion stings annually cause 2000–3000 deaths as they can lead to the respiratory and/or cardiovascular complications. Pathogenesis of lung damage under scorpion envenomation is often comprehensive. Respiratory failure can have a cardiogenic origin, associated with venom neurotoxin action. However, some venom components can stimulate pro-inflammatory signaling cascades followed by cytokines synthesis, recruit and activate immune cells, participating in the inflammatory response in lung injury. Scorpions of the *Leiurus* genus ("deathstalker") are one of the most dangerous *Arthropoda*. To date, 22 species of this genus have been described, but the venom composition and the mechanisms of tissues damage under envenomation have been studied to some extent only for *L. quinquestriatus*, *L. hebraeus*, and *L. abdullahbayrami*. Scorpions of L. *macroctenus* species are expected to be very hazardous, but the possibility of their venom cause inflammation in the lung tissue has not been investigated to date. Therefore, in this study, we focused on evaluating the levels of cytokines and their regulators – transcription factors (HIF-1α and NF-κB) and growth factors (FGF-2, VEGF, and EGF) – in rat lung homogenates after *L. macroctenus* envenomation. The results revealed a decrease in the levels of most pro-inflammatory cytokines (IL-6, IL-8, IL-1β and TNF-α) with simultaneous rise in the content of both anti-inflammatory cytokines (IL-4 and IL-10) and interferon-γ. Furthermore, the levels of all researched transcription factors and growth factors were shown to be increased too. The detected changes peak occurred at 24 h, whereas a tendency towards all indicators values normalization was observed in 72 h after venom injection. Thus, our results did not reveal signs of a classic inflammatory process in the lungs of rats injected with *L. macroctenus* venom. However, the obtained data indicate venom influence both on cytokine profile and on their regulators content in the rat lungs, which is a feature of certain alterations in the innate immune response, caused by studied venom components. But, the mechanisms of the changes we found require additional researches.

## Highlights

• *L*. *macroctenus* envenomation cause decrease in the levels of most pro-inflammatory cytokines.
• Levels of transcription factors and growth factors increase after *L. macroctenus* envenomation.
• *L*. m*acroctenus* venom rise the content of anti-inflammatory cytokines and interferon-γ.
• Peak of changes occurred at 24 h after *L. macroctenus* venom injection.
• Components of the venom of the scorpion *L. macroctenus* activate the innate immune response.

## 1 Introduction

Nearly 1.5 million scorpion envenomations are registered annually in the world (Ahmadi et al., 2020). These animals are represented by more than 2200 species and belong to the Phylum *Arthropoda*, Class *Arachnida* and Order *Scorpiones* (Tobassum et al., 2020). The vast majority of scorpion species harmful to humans are members of the *Buthidae* family. Although they are the inhabitants of tropical and subtropical countries, scorpion stings cases are also noted in Turkey, Spain, France, China, and Mongolia (Ahmadi et al., 2020; Moradzadeh Roozbehani et al., 2023). The most common causes of scorpion envenomation are the domestic incidents in the rural areas. But the uncontrolled expansion of harmful scorpion species, climate changes, and rapid urban expansion along with poor city sanitation have led to an increase in the stings number. Therefore, in developing countries scorpion envenomation still remains an urgent public health problem (Godoy et al., 2021).

Mild and moderate envenomations are characterized by local pain, erythema and edema at the sting site, nausea, vomiting, fever, hypersalivation, restlessness, abnormally rapid breathing and increased heart rate, and usually don't carry a risk to life (Ward et al., 2018). Such life-threatening conditions as acute respiratory distress syndrome, cardiac dysfunction, pulmonary edema, cardiogenic shock, and multiple organ failure can develop in severe envenomations and lead to death without medical intervention (Ryan et al., 2021). Most often, a scorpion sting causes only local symptoms (81% of cases), approximately 5% of reported cases are severe and only 0.3% of severe cases are fatal (Tobassum et al., 2020).

Scorpion venom is a mix containing disulfide-bridged and non-disulfide-bridged peptides (DBPs and NDBPs, respectively), enzymes, nucleotides, lipids, mucoproteins, biogenic amines, serotonin, histamine, free amino acids, inorganic salts and other components (Tobassum et al., 2020; Ghosh et al., 2019). Peptides make up about 5% of the dry weight of scorpion venom and are the most studied (Hmed et al., 2013). DBPs are neurotoxins targeting voltage-gated ion channels (Abdoon et al., 2006; Gómez et al., 2019). Na^+^-channel toxins involve long-chain peptides and are divided into α-toxins, inhibiting Na^+^-channel inactivation, and β-toxins, causing their inactivation. Toxins targeting K^+^-, Ca^2+^-, and Cl^−^-channels belong to short-chain peptides. Neurotoxins can impact on neurotransmitter, hormonal and cytokine secretion, water-salt balance, and blood pressure resulting in the cardiovascular, pulmonary and gastrointestinal complications (Ahmadi et al., 2020; Hmed et al., 2013; Petricevich, 2010; Gunas et al., 2023a). NDBPs are small amphipathic peptides that nonspecifically interact with membranes, exhibiting antibacterial, antifungal or antiviral effects. Some of them have bradykinin-potentiating, immunomodulatory or other activities (Abdoon et al., 2006). Enzymes and their inhibitors are also present in scorpion venom. Hyaluronidases and metalloproteinases cleave extracellular matrix (ECM) components, accelerating toxin diffusion. Phospholipases are hemolytic agents destroying cell membranes. Metallo- and serine proteases activate latent forms of toxins and endogenous signaling molecules, as well as modulate cytokine production (Petricevich, 2010). Thus, different mechanisms initiated by venom components could be equally important for the development of scorpion envenomation signs, including inflammatory response in injured tissue, as some venom components can have immunomodulatoty effects.

Scorpions of the *Leiurus* genus ("deathstalker") are one of the most dangerous *Arthropoda* (Koç et al., 2016). Until recently, this genus was considered to be monospecific for *L. quinquestriatus*, but it has undergone significant taxonomic reclassification in the last few years and includes 22 species now, each of which has a fairly clear geographical range (Ward et al., 2018; Borges and Lomonte, 2024). *L. macroctenus* differs from other *Leiurus* genus species by specific morphometric and morphological features; it was described in 2014 by Lowe et al. with following confirmation of its existence at the genetic level (Lowe et al., 2014a; Alqahtani and Badry, 2020). To date, this scorpion venom composition has not been established. The studied venoms of related species are generally characterized by the presence of the components described above (Borges and Lomonte, 2024). However, it should be noted that the venoms of even closely related species can differ significantly in their quantitative and sometimes qualitative composition (Tobassum et al., 2020).

Currently, little is known about the role of inflammatory phenomena in the pathogenesis of *L. macroctenus* envenomation. There are also no data on the pro- and anti-inflammatory cytokines levels in the damaged organs under this condition. Taking into account the fact that lung injury is a common symptom in the severe scorpion envenomings, the aim of this work was to determine the content of cytokines and their regulators – transcription factors and growth factors - in the rat lung homogenates after *L. macroctenus* venom injection.

## 2 Materials and methods

### 2.1 Scorpion collection and maintenance

To obtain the scorpion venom, ten mature L. macroctenus specimens were taken from the wild on the territory of Oman, identified by Mark Stockmann according to morphological features (Lowe et al., 2014b) and kept in Ibbenbüren private collection (Germany). Scorpions were maintained separately from each other in transparent plastic containers (10 × 5 × 5 cm) filled with sand (Exo Terra "Desert Sand") by 1 cm. Drinking bowls with weekly refilled distilled water were placed in the center of each box. A stable temperature (25◦C–35 °C), humidity of 50–60% as well as natural lighting regime were maintained in the containers. Appropriate aeration conditions were reached by numerous holes in the boxes. *Shelfordella lateralis* (Turkestan cockroach) was used as food - each scorpion was offered 1 cockroach for food every week. Feeding using only cockroaches was used for at least a year. In the case of food refusal, after 2 days, the cockroach was removed from the container. Once a month, the sand was cleaned from the remains of cockroaches.

### 2.2 Venom collection

The procedure of venom collection was performed according to Ozkan and Filazi's electrostimulation method modified by Yaqoob et al. (Ozkan and Filazi, 2004; Yaqoob et al., 2016). After scorpion fixation, electrodes were pointed to cephalotorax and telson, and an electric current with an intensity of 24 V was applied for 5 s to the base of the telson, while the opposite edge of the telson was directed to the sterile phial. The number of electrode-scorpion contacts varied up to 10 depending on an amount of collected venom. The collected venom samples were stored at −20 °C. Venom milking procedure was performed every 2 weeks.

### 2.3 Venom injection and rat lungs homogenization

Laboratory albino male rats were kept in the accredited vivarium of the Educational and Scientific Center "Institute of Biology and Medicine" of Taras Shevchenko Kyiv National University in accordance with the "Standard Rules for Organizing, Equipping and Maintaining Experimental Biological Clinics (vivariums)". Animals kept on a standard diet under temperature 20–24 °C, humidity - 30–70 %, 12-h light day. Rats selected for the experiment (180 g ± 3 g) were subjected to a veterinary examination with following dividing into groups, weighing, numbering and marking accordingly.

The experimental group consisted of 60 animals injected intramuscularly with 0.5 ml venom solution (28.8 μg/ml; LD50 = 0.08 mg/kg), previously dissolved in saline solution (0.9 %), whereas the control group animals (13 rats) were injected with 0.5 ml saline solution (0.9 %) alone.

The rats were euthanized with carbon dioxide inhalation method. Rat lungs isolation and homogenization were performed at 4 °C, just after euthanasia. 50 mM Tris-HCl (pH 7.4) buffer with 140 mM NaCl and 1 mM EDTA addition was used for homogenization, and buffer amount (in grams) was five times higher than mass of isolated organs. Obtained crude homogenate was centrifuged (600 g, 15 min) with further supernatant collection and recentrifugation at 15 000 g for 15 min to get rid of nuclear and mitochondrial fractions. Obtained homogenate aliquots were frozen in liquid nitrogen.

### 2.4 Quantifcation of the parameters in rat lung homogenates

The levels of pro- and anti-inflammatory cytokines (interleukins (ILs) IL-1β, IL-4, IL-6, IL-8, IL-10, tumor necrosis factor-α (TNF-α), interferon-γ (IFN-γ)) as well as their regulators (α-subunit of hypoxia-inducible factor-1 (HIF-1α), nuclear factor-kappa B (NF-κB), fibroblast growth factor-2 (FGF-2), vascular endothelial growth factor (VEGF), epidermal growth factor (EGF)) content in lung homogenates were done by enzyme-linked immunosorbent assay (Commercial ELISA kits, Biotrak ELISA System, Healthcare, USA) according to the standard instructions for soluble proteins (Crowther, 2000). The samples of homogenate were diluted to 1 μg/ml with 0.05 M Tris-HCl buffer (pH 7.4) and incubated overnight at 4 °C in sterile ELISA plates wells with following washing of the wells with immobilization buffer to remove unbound antigen. To avoid nonspecifc binding, incubation with 5% non-fat dry milk solution for 1 h at 37°С was used. After that, plates were washed again with buffer containing 0.1% Tween-20, with further incubation with the corresponding primary antibodies (Santa Cruz Biotechnology, USA) (1 : 3000) for 1 h at 37 °C. Next, plates were washed with buffer containing 0.1% Tween-20 and incubated with horseradish peroxidase-conjugated secondary antibodies (Bio-Rad, USA) (1 : 3000) for 1 h at 37 °C. After that, wells were washed with 0.1% Tween-20 buffer and incubated with 0.4 mg/ml o-phenylenediamine (Sigma-Aldrich, USA), diluted in 0.05 M citrate-phosphate buffer, in the presence of 30% H_2_O_2_ to visualize the reaction. The reaction was stopped 10 min later by adding 100 μl 1 M H_2_SO_4_. Optical density of the samples was measured on a microplate reader (μQuantTM, BioTek Instruments, Inc) at wavelength 492 nm.

### 2.5 Statistical analysis of results

Data entry and analysis were performed using MS Excel (MS Office) and StatSoft Statistica ver.8.0 for Windows. After testing for normality (by Shapiro-Wilk), a one-way analysis of variance (ANOVA) was used to compare the means among different groups. Differences were statistically significant when p < 0.05.

### Ethical approval

All experiments on animals were performed in the compliance with international principles of the European Convention for the protection of vertebrate animals used for experimental and other scientific purposes (Strasbourg, 1986). The study was approved by the Ethical Committee of Taras Shevchenko National University of Kyiv (protocol N◦2 approved August 19, 2021).

## 3 Results

### 3.1 Cytokine profile analysis in rat lungs homogenates

**Fig. 1:**
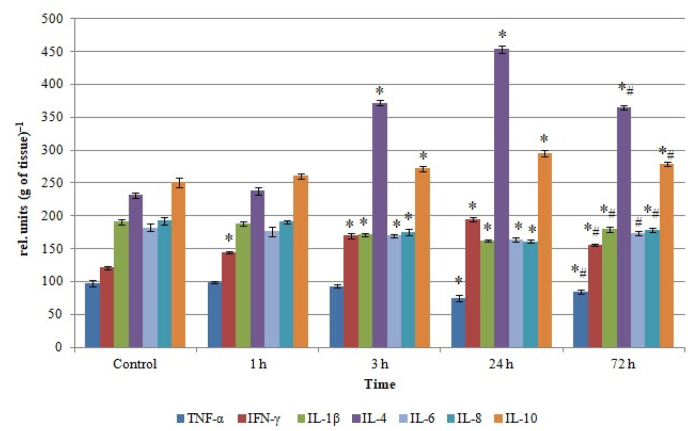
Cytokine profile of rat lung homogenates after *Leiurus macroctenus* envenomation. Results are presented as mean ± SEM (n = 5). ∗ p < 0.05 vs. control, #p < 0.05 vs. 24th h.

It was established that the levels of all studied cytokines (with the exception of IFN-γ) in rat lung homogenates did not differ significantly from the control values 1 h after *L. macroctenus* venom injection (Fig. 1). But multidirectional changes (р<0.05) in the pro- and anti-inflammatory cytokines levels were found in the next periods – from 3rd to 24th h after envenomation. In particular, our results demonstrated a moderate decrease in the content of pro-inflammatory IL-6 (by 7% and 10% in 3 and 24 h after venom injection, respectively), IL-8 (by 9% and 16%) and IL-1β (by 10 % and 15%), as well as a more notable (by 23%) lowering in the TNF-α level in 24 h after envenoming.

Instead, a considerable rise in the content of anti-inflammatory IL-4 (by 1.6 and 2 times in 3 and 24 h after envenomation, respectively) and IL-10 (by 8% and 17%) was noticed. The content of pro-inflammatory IFN-γ was also increased starting from the 1st h after venom injection (by 18%, 40% and by 1.6 times, respectively, in 1, 3 and 24 h). At the same time, a tendency towards normalization of all studied indicators levels was revealed in 72 h after envenomation, namely, elevation in the content of pro-inflammatory IL-6 (by 6%), IL-8 (by 11%), IL-1β (by 10%) and TNF-α (by 14%) and decline in the levels of anti-inflammatory IL-4 (by 20%) and IL-10 (by 7%), as well as pro-inflammatory IFN-γ (by 20%) comparing to the values of the corresponding indicators at 24 h. As a result, IL-6 content in the rat lung homogenates did not significantly differ from the control in 72 h after venom injection, while IL-8, IL-1β and TNF-α levels remained lower (by 7%, 7% and 12%, respectively), and IL-4, IL-10 and IFN-γ content – higher (by 1.6 times, by 11% and 29%, respectively) comparing to the control values.

### 3.2 Quantifcation of HIF-1α and NF-κB in rat lungs homogenates

**Fig. 2:**
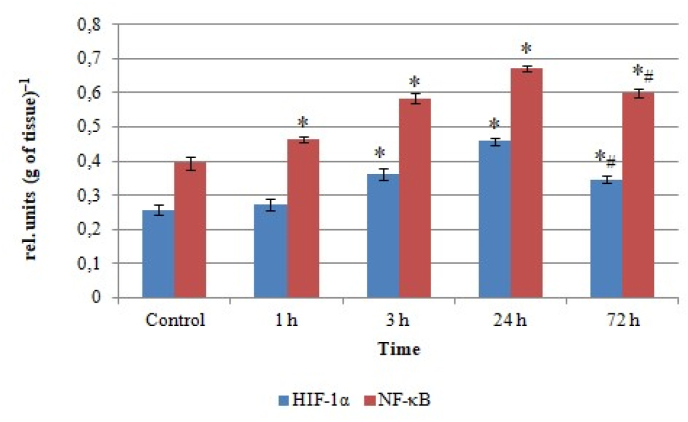
HIF-1α and NF-κB content in rat lung homogenates after *Leiurus macroctenus* envenomation. Results are presented as mean ± SEM (n = 5). ∗ p < 0.05 vs. control, #p < 0.05 vs. 24th h.

Transcription factors including HIF-1 and NF-κB are important regulators of cytokine production. Their content analysis in the rat lung homogenates after *L. macroctenus* venom injection revealed a gradual increase in the values of the corresponding indicators with peak in 24 h after envenomation (Fig. 2).

An increase in NF-κB content was observed at all investigated time points (by 18%, 48% and by 1.7 times, p < 0.05, in 1, 3 and 24 h after venom injection, respectively), whereas rising in the levels of HIF-1α (which is the hypoxia-regulated HIF-1 subunit) started only in 3 h and amounted to 40% (3rd h) and 1.8 times (24th h). In 72 h after envenomation, the studied indicators levels declined comparing to 24 h (by 11% and 24% for NF-κB and HIF-1α, respectively), but still exceeded the control values by 1.5 times (NF-κB) and by 35% (HIF-1α).

### 3.3 Growth factor content determination in rat lungs homogenates

**Fig. 3:**
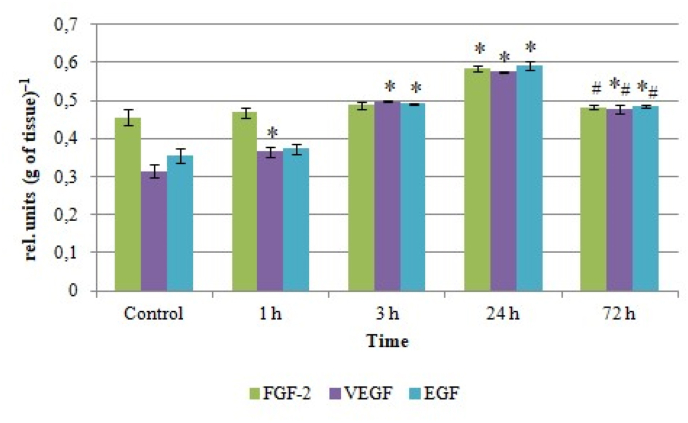
Growth factor content in rat lung homogenates after *Leiurus macroctenus* envenomation. Results are presented as mean ± SEM (n = 5). ∗ p < 0.05 vs. control, #p < 0.05 vs. 24th h.

Similar changes were observed in the levels of other cytokine production regulators such as growth factors FGF-2, VEGF and EGF. It was shown, that the rise in the FGF-2 content began later, only in 24 h after venom injection, and amounted to 20% of the control value, and in 72 h after envenoming, this indicator level did not significantly differ from that in the control group (Fig. 3).

The most noticeable changes related to VEGF, which levels increased by 16% (1st h), by 1.6 times (3rd h) and 1.8 times (24th h); in 72 h after envenomation, the VEGF content diminished by 17% comparing to 24th h, but still exceeded the control values by 1.5 times. The increase in EGF levels, in turn, amounted to 38% (3rd h), 1.7 times (24th h) and 36% (72nd h; but simultaneously EGF content decreased by 18% comparing to the peak at 24th h).

## 4 Discussion

When entering the body, venom components can diffuse to different tissues causing their damage. Venom hyaluronidases and metalloproteinases degrade ECM components, accelerating toxin distribution. Some peptide toxins, as well as metallo- and serine proteases, can activate inflammatory reaction through stimulation of pro-inflammatory signaling cascades followed by cytokines synthesis, recruitment and activation of immune cells, whereas other may have immunosuppressive action and down-regulate inflammatory response. Lung injure accompanied by inflammation have been documented in cases of severe and fatal envenoming by *L. abdullahbayrami* and *L. quinquestriatus* related to *L. macroctenus* (Borges and Lomonte, 2024; Fereidooni et al., 2023). The toxic effect of the poison of L. macroctenus on the kidneys was proven in the study of Matkivska R. with co-authors. In the work, authors described imbalance in pro- and anti-inflammatory cytokines levels during envenomation with significantly increased levels of TFs HIF-1α and NF-κB, and GFs EGF, FGF-2, and VEGF, as well as IFN-γ (Matkivska et al., 2024). There are limited data on this scorpion venom influence on the lungs (Gunas et al., 2023a, 2023b). Thus, evaluation of cytokines and their regulators content in the lungs of rats injected by *L. macroctenus* venom is important for studying the mechanisms of this potentially dangerous scorpion venom action on lung tissue and inflammation response involvement in these mechanisms.

### 4.1 Cytokine profile analysis

Our study results revealed multidirectional changes in the levels of pro- and anti-inflammatory cytokines in rat lung homogenates after *L. macroctenus* venom injection, namely, a decline in the content of IL-1β, IL-6, IL-8, and TNF-α and an increase in IL- 4, IL-10, as well as in IFN-γ levels. TNF-α and IL-4 levels changed the most, and the detected changes peak was shown in 24 h after venom injection, while in 72 h the values of the studied parameters tended towards normalizing, approaching the control values.

Cytokines released by immune cells regulate the immune response, induce tissue damage, and mediate inflammatory response complications. Pro-inflammatory cytokines IL-1, IL-6 and TNF-α are responsible for effective protection against exogenous pathogens, but under overproduction they can be harmful. On the contrary, the anti-inflammatory cytokines IL-4 and IL-10 that are critical for repressing the acute inflammatory process, under excessive releasing can suppress the body immune function. Therefore, the balance between pro- and anti-inflammatory cytokines is important for maintaining homeostasis in the system. A number of studies report the high serum levels of pro-inflammatory cytokines both in humans bitten by scorpions and in experimental animals envenomed by *Tityus serrulatus*, *Centruroides noxious*, *Buthus occitanus* and *L. quinquestriatus* (Moradzadeh Roozbehani et al., 2023; Gunas et al., 2023a; Razi et al., 2015; Saidi et al., 2013). There is also evidence of an increase in IL-1β content in mice lungs 4 h after intraperitoneally injection of *T. serrulatus* venom, as well as a rise in the production of pro-inflammatory cytokines by macrophages (in vitro model) with the highest levels of IL-1α, IL-1β and TNF-α 12 h after venom administration, IL-6 - 24 h and IFN-γ - 72 h after envenomation (Zoccal et al., 2016; Petricevich and Lebrun, 2005). However, our results which did not reveal changes in the cytokine profile that would indicate the inflammation development in the lungs, do not contradict these data. Indeed, our study concern the cytokine content in lung homogenates, and the serum cytokine profile does not reflect that of tissues. Moreover, rats were used for animal model creation in our research, as well as other scorpion species venom with other composition, and cytokine levels were measured at other time points, namely, 1, 3, 24 and 72 h after the venom injection.

Along with this, the results obtained earlier in our laboratory, showed a rise in total proteolytic activity, activities of metallo- and serine proteases, as well as an increase in matrix metalloproteinases (MMPs) and medium-mass molecules (MMMs) content in the lungs of rats envenomed by *L. macroctenus*, and these changes were observed starting from the 1st h with a peak at 24 h after venom injection (Gunas et al., 2023a, 2023b). It is known that pro-inflammatory cytokines stimulate MMPs genes expression, promoting proteolysis. Thus, it can be assumed that an increase in pro-inflammatory cytokines content could occur earlier, within 1st h after venom administration, which could not be detected in our study because we did not examine these molecules content during this time period. Such fulminant inflammation development with an increase in both pro- and anti-inflammatory cytokines content within 30 min after venom injection was shown in experiments on mice envenomed by *Androctonus australis hector* scorpion (*Buthidae* family) (Adi-Bessalem et al., 2008). In this context, a rise in anti-inflammatory cytokines content we revealed may indicate a change in the phenotype of the predominant population of macrophages in the lung tissue, since alterations in cytokines expression are important indicators of this process. M1 phenotype macrophages produce IL-1β, IL-6, TNF-α, and IFN-γ, exacerbating inflammation, and efficiently phagocytize damaged tissue debris. Anti-inflammatory M2 phenotype macrophages secrete IL-4 and IL-10, suppressing inflammatory reactions, and also synthesize a number of growth factors, being involved in extracellular matrix remodeling, angiogenesis and regenerative processes (Chávez-Galán et al., 2015). Elevated IFN-γ levels contribute to macrophages polarization into M1 phenotype, while high IL-10 content causes M1-to-M2 macrophages transition (Cheng et al., 2021). But the stimuli promoting macrophages polarization into different phenotypes often coexist, so macrophages can simultaneously express genes of both phenotypes, showing a more pro-inflammatory (M1-like) phenotype in the early stage of inflammation, and a more anti-inflammatory (M2-like) phenotype in the late stage (Strizova et al., 2023). According to this hypothesis, the decrease in the pro-inflammatory cytokines content in the lung tissue we revealed may be a consequence of their natural antagonists action such as IL-4 and IL-10, which content rise in the lung tissue. In particular, IL-4 is able to block IL-1, IL-6, IL-8, TNF-α synthesis, whereas IL-10 inhibits formation of IFN-γ, TNF-α, IL-1β, IL-2, IL-6, IL8 and IL-12 (Moradzadeh Roozbehani et al., 2023), (Gunas et al., 2023a). IFN-γ content also was shown to be increased in our study. Although it belongs to pro-inflammatory cytokines, but shows pleiotropic properties and, like anti-inflammatory cytokines, is able to prevent excessive immune system activation and tissue damage.

However, we can also suggest, that this venom cause no inflammation, as some of its components may have immunosuppressive action related to the modulation of various signaling pathways, suppression of recruitment/activation of immune cells and inhibition of pro-inflammatory cytokines release. In particular, since different types of voltage-gated potassium channels are involved in immune cells regulation, some K^+^-channel toxins from short-chain DBPs group can suppress their activation and secretion of pro-inflammatory cytokines. Toxins with such effects were isolated from the venoms of scorpions from the *Buthus*, *Mesobuthus*, *Parabuthus*, *Androctonus*, *Lychas*, *Leiurus*, *Tityus*, and *Centruroides* genera (Ahmadi et al., 2020; Abdoon et al., 2006). In this context, proteolysis intensification and a rise in MMPs content previously detected in our laboratory may be linked with the mechanisms that do not involve pro-inflammatory cytokines, but are related to the venom components action on signaling pathways and/or directly on proteolytic enzymes. Particularly, venom proteases are able to hydrolyze ECM components, or act indirectly, activating latent forms of MMPs, which then degrade matrix proteins. And some venom peptides and/or enzymes potentially can modulate signaling pathways, affecting MMPs gene expression. The increase in MMMs content in the lungs of rats envenomed by *L. macroctenus* was also found in our previous studies (Gunas et al., 2023b). High MMMs levels are usually associated with a pro-inflammatory state, as these compounds can act as endogenous DAMPs. However, this study showed that an increase in their content in the lungs is not accompanied by a simultaneous rise in the level of pro-inflammatory cytokines. It may be related to the presence of venom components suppressing inflammation by affecting cytokine release from immune cells and/or signaling pathways, stimulated by DAMPs. In addition, since the spectrum and properties of the compounds that make up MMMs fraction under this venom action are unknown, we cannot claim that they necessarily have pro-inflammatory properties.

Thus, *L. macroctenus* venom may potentially have components with both pro- and anti-inflammatory properties, and these components may cause complex effects on lung tissue. Our data revealed the changes in cytokine profile of the rat lungs after this venom injection indicating certain alterations in the innate immune response. However, our results needs in additional investigations. Analysis of serum cytokine content and study of lung cellular infiltrate at different time points after venom injection can help to reveal the involvement of pro- and anti-inflammatory pathways as well as allergic processes in the envenomation mechanisms. Examination of crude venom and its toxins effects on isolated immune cells may be helpful to detect their potential impact on both cytokine release by immune cells and signaling pathways stimulated by DAMPs. Histological studies of the lung tissue could be also useful.

### 4.2 HIF-1α and NF-κB content during Leiurus macroctenus envenomation

In order to study the possible mechanisms of the rat lung homogenate cytokine profile changes we discovered, the transcription factors NF-κB and HIF-1α levels were further analyzed, as these proteins are important regulators of cytokine biosynthesis. We found a significant rise in their content after venom injection, which peak coincided in time with the maximum changes detected in the cytokines levels (24 h). Both transcription factors content increased to about the same extent, but the increase in HIF-1α level started later than one of NF-κB.

Although the mechanisms by which the immune system detects venom components and initiates inflammation, as well as the signaling pathways that regulate the immune response, remain unknown, a number of studies indicates the implication of pattern recognition receptors (PRRs) located on innate immune system cells, such as Toll-like receptors (TLRs), in these processes. While TLRs are usually activated by ligands of bacterial or viral origin (pathogen-associated molecular patterns) or by endogenous compounds (damage-associated molecular patterns), recent studies indicate that TLR2 and TLR4 can also recognize non-microbial ligands, including certain venom components (so-called venom-associated molecular patterns) with following stimulation of pro-inflammatory pathways (Martin-Eauclaire et al., 2019; Malaque et al., 2015; Reis et al., 2019). For instance, it was found that *T. serrulatus* venom, interacting with TLR2, TLR4 and CD14 of macrophages, activates these cells and through NF-κB- and AP-1-dependent signaling pathways promote them to generate the cytokines and lipid mediators (Ryan et al., 2021; Zoccal et al., 2019). At the same time, an increase in NF-κB content in rat lung homogenates under scorpion envenomation can also be caused by the reactive oxygen species action. They are strongly produced in the envenomed animal tissues and can cause an increase in NF-κB expression via oxidative modulation of a number of signaling pathways (Reis et al., 2019).

The cellular response to hypoxia is mediated by a family of hypoxia-inducible transcription factors (HIFs). HIF-1 is a heterodimer consisting of the oxygen-sensitive subunit HIF-1α and HIF-1β (Zhang et al., 2016). HIF-1α is expressed in almost all cells of innate and adaptive immunity. Under normoxic conditions, HIF-1a is subject to ubiquitin-dependent proteasomal degradation, but under conditions of low oxygen tension, it dimerizes with HIF-1β and upregulates genes induced by hypoxia (Palazon et al., 2014). Respiratory and cardiovascular complications of scorpion envenomation lead to acute hypoxia and rapid deterioration of respiratory status, which may contribute to HIF-1α stabilization and HIF-1 activation (Feola et al., 2020). Along with this, HIF-1 is also involved in the regulation of the immune response. Due to HIF-1, hypoxia significantly changes the gene expression profile of macrophages accumulating in hypoxic areas, inducing their production of many pro-inflammatory cytokines and chemokines genes (Imtiyaz and Simon, 2010). In turn, pro-inflammatory cytokines such as TNF-α, IL-1β and IL-6, as well as growth factors and bacterial products can induce HIF under normoxic conditions (TNF-α and IL-1β – through NF-κB activation) (Palazon et al., 2014; Imtiyaz and Simon, 2010). There is also evidence of linking HIF-1α with TLR4 and inflammation development. As mentioned above, in macrophages TLR4 can be activated by scorpion venom components, and downstream signaling leads to HIF-1α accumulation, which along with NF-kB activation is important for TLR4-dependent expression of pro-inflammatory cytokines (Zhang et al., 2016). On the other hand, HIF-1α upregulates TLR4 expression under hypoxic conditions (Imtiyaz and Simon, 2010).

### 4.3 FGF-2, VEGF and EGF levels after Leiurus macroctenus venom injection

Our results also revealed an increase in the content of other regulators of cytokine production - growth factors FGF-2, VEGF and EGF - in rat lung homogenates under *L. macroctenus* envenomation. The largest changes were found in the levels of VEGF, the smallest - for FGF-2 content, and the detected changes time frames generally coincided with those for the other studied indicators.

It is known that HIF1-α, which content rise during envenomation was revealed in the results of this study, is one of FGF-2 and VEGF expression activators (Akhtar et al., 2021). Moreover, VEGF levels rise can be also a sign of M1-to-М2 macrophages transition, as M2 phenotype macrophages generate growth factors which promote blood vessel development and cellular proliferation (Strizova et al., 2023).

In addition, important regulators of growth factors and cytokines content are represented by MMPs. And, as noted earlier, MMPs content in the lungs of rats envenomed by *L. macroctenus* increased starting at 1 h after venom injection with a peak at 24 h (Gunas et al., 2023a). It is known that MMPs affects chemokines processing and therefore play a key role in their functions regulation, influencing the leukocytes recruitment to the inflammation site (de Almeida et al., 2022). By degrading ECM, these enzymes release growth factors and cytokines non-covalently bound to ECM components, which increases their bioavailability. In particular, VEGF latent form is non-covalently bound to heparan sulfates and is released during ECM proteolysis. As the number of such examples is constantly increasing, growth factors and cytokines (including IL-1β, IL-8 and TNF) processing is now suggested as one of the main functions of MMPs in vivo (Löffek et al., 2011). In turn, MMP expression is regulated by the wide range of cytokines, chemokines, and growth factors that act via transcription factors activation including AP-1, NF-κB and other (de Almeida et al., 2022; Sternlicht and Werb, 2001).

Moreover, ECM integrity can be violated by venom enzymes, such as hyaluronidase, as well as metallo- and serine proteases, and these mechanisms can be also involved in the modulation of growth factors production (Ahmadi et al., 2020; Koç et al., 2016; Gunas et al., 2023b).

Therefore, the rat lung homogenate cytokine profile under *L. macroctenus* envenomation is characterized by declining the content of most pro-inflammatory cytokines (IL-6, IL-8, IL-1β and TNF-α) and rising the levels of anti-inflammatory interleukines (IL-4, IL-10) and pleiotropic IFN-gamma. The detected changes are maximally pronounced in 24 h after venom injection, while in 72 h the cytokine content of both groups trends toward normalization. In addition, HIF-1α and NF-κB levels as well as FGF-2, VEGF and EGF content, which are considered to be the important regulators of cytokine production and release, are increased in rat lung homogenates during envenomation, and the time characteristics of this rise are similar to the dynamics of the detected changes in the cytokines content.

## 5 Conclusions

Thus, our results did not reveal signs of a classic inflammatory process in the lungs of rats injected with *L. macroctenus* venom. However, the obtained data indicate venom influence both on cytokine profile and on their regulators content in the rat lungs, which is a feature of certain alterations in the innate immune response, caused by studied venom components. But, the mechanisms of the changes we found require additional researches.

## Authors’ contributions

Valery Gunas was in charge of conceptualization; Tetiana Vovk was in charge of data curation and visualization; Nataliia Raksha was in charge of formal analysis and validation; Valery Gunas was in charge of investigation, project administration; Tetiana Halenova was in charge of writing the review, and editing; Oleksiy Savchuk was in charge of methodology and writing the original draft; Igor Gunas was in charge of resources; Tatyana Synelnyk handled software; and Oleksandr Maievskyi was in charge of supervision.

## Ethical approval

All experiments on animals were performed in the compliance with international principles of the European Convention for the protection of vertebrate animals used for experimental and other scientific purposes (Strasbourg, 1986). The study was approved by the Ethical Committee of Taras Shevchenko National University of Kyiv (protocol N◦2 approved August 19, 2021).

## CRediT authorship contribution statement

**Valery Gunas:** Conceptualization. **Oleksandr Maievskyi:** Supervision. **Tatyana Synelnyk:** Software. **Nataliia Raksha:** Validation, Formal analysis. **Tetiana Vovk:** Visualization, Data curation. **Tetiana Halenova:** Writing – review & editing. **Olexiy Savchuk:** Writing – original draft, Methodology. **Igor Gunas:** Resources.

## Declaration of competing interest

The authors declare that they have no known competing financial interests or personal relationships that could have appeared to influence the work reported in this paper.

## Data Availability

Data will be made available on request.
